# Large Range of a High-Precision, Independent, Sub-Mirror Three-Dimensional Co-Phase Error Sensing and Correction Method via a Mask and Population Algorithm

**DOI:** 10.3390/s24010279

**Published:** 2024-01-03

**Authors:** Dequan Li, Dong Wang, Jingquan Li

**Affiliations:** 1 Space Optics Department, Changchun Institute of Optics, Fine Mechanics and Physics, Chinese Academy of Sciences, Changchun 130033, China; 2 Electrical and Electronic Teaching and Research Section, Basic Department, Aviation University of Air Force, Changchun 130022, China

**Keywords:** segmented mirrors, model-free online correction, co-phase error, population optimization algorithm

## Abstract

The emergence of segmented mirrors is expected to solve the design, processing, manufacturing, testing, and launching of space telescopes of large apertures. However, with the increase in the number of sub-mirrors, the sensing and correction of co-phase errors in segmented mirrors will be very difficult. In this paper, an independent three-dimensional method for sub-mirror co-phase error sensing and correction method is proposed. The method is based on a wide spectral modulation transfer function (*MTF*), mask, population optimization algorithm, and online model-free correction. In this method, the sensing and correction process of each sub-mirror co-phase error is independent of each other, so the increase in the number of sub-mirrors will not increase the difficulty of the method. This method can sense and correct the co-phase errors of three dimensions of the sub-mirror, including piston, tip, and tilt, even without modeling the optical system, and has a wide detection range and high precision. And the efficiency is high because the sub-mirrors can be corrected simultaneously in parallel. Simulation results show that the proposed method can effectively sense and correct the co-phase errors of the sub-mirrors in the range [−50*λ*, 50*λ*] in three dimensions with high precision. The average RMSE value in 100 experiments of the true co-phase error values and the experimental co-phase error values of one of the six sub-mirrors is 2.358 × 10^−7^*λ*.

## 1. Introduction

The light-gathering capability and resolution of a telescope are directly related to the size of its primary mirror aperture, so both space telescopes and ground-based telescopes are constantly developing in the direction of large apertures. However, the increase in the size of the primary mirror has brought unprecedented challenges to the design, processing, manufacturing, testing, and launching of space telescopes. The emergence of segmented telescopes provides a solution to the above problems [1,2,3].

In order to make the overall image quality of the segmented telescope close to the diffraction limit, it is necessary to ensure high co-phase accuracy between the sub-mirrors. In this process, the co-phase errors of each sub-mirror need to be detected first and then corrected by a high-precision displacement adjusting mechanism [4,5,6]. At this stage, there are many commonly used wavefront sensing methods, some of which depend on hardware devices [7,8,9,10,11,12,13,14], while others are based on images [15,16,17,18].

The hardware-based method has the advantages of high sensitivity and strong anti-interference ability, but the optical path is generally more complex, and the use cost is high. The image-based method has the advantages of a simple optical path and simple application conditions, but it requires a lot of computation or depends on the accurate registration of the simulation model and real model.

In reference [19,20,21], the authors placed a mask on the pupil surface of the optical system and realized the decoupling and detection of the piston error of each sub-mirror based on wide spectrum *MTF*. This method has low hardware requirements, a large detection range, and high accuracy.

In this paper, a sub-mirror three-dimensional co-phase error sensing and correct method with a large range and high accuracy is proposed based on the above method. The optical system mask configuration in this method is the same as the above method. The main idea is to use the *MTF* sub-peak heights as the evaluation function of the three-dimensional co-phase error of the sub-mirror. After correcting the co-phase error of the sub-mirror by real-time correction method, the reward function is calculated to judge the quality of the adjustment amount, which is each solution in the cuckoo search optimization algorithm.

This method retains the advantage of the original method in that the detection of the co-phase error of each sub-mirror is independent of each other. And it does not rely on the strict correspondence between the *MTF* value and the co-phase error value nor does it require modeling of the optical system. Therefore, the conditions of use are simpler, which are not available in the original method. In addition, since the input of the evaluation function in this paper is the co-phase error of each sub-mirror, the optimization dimension of the population algorithm is 3. Therefore, the optimization difficulty of the optimization algorithm remains not too high even if the solution range is large. The process of detecting and correcting the co-phase errors of each sub-mirror is independent of each other. Therefore, increasing the number of sub-mirrors only needs to change the shape of the mask according to the rules, which will not increase the difficulty of the algorithm. Since the co-phase error correction process of each sub-mirror can be carried out simultaneously without interfering with each other, the method has high efficiency.

This paper is structured as follows: In Section 2, we will describe the optical imaging system we used. In Section 3, we will explain the proposed method in detail. In Section 4, we will carry out the relevant simulation experiments and illustrate the relevant experimental results. The Conclusions are provided in Section 5.

## 2. Phasing Piston Error in Segmented Telescopes by Mask

**Figure 1:**
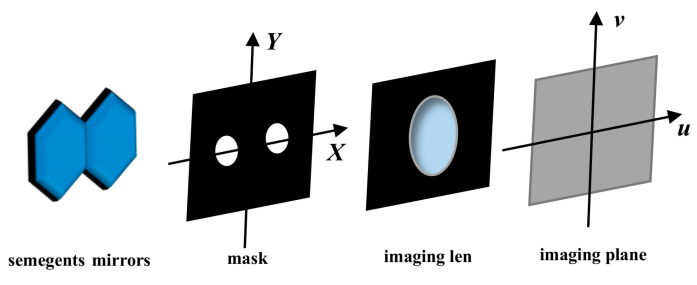
The schematic diagram of segmented mirrors and mask configuration.

As shown in Figure 1, assuming that a mask with two sparse sub-pupils is set up at the exit pupil surface of the segmentation system, the reflected sub-waves of the two segments are sampled, and the incident light is a broad spectrum with a center wavelength $\lambda_{0}$ and a spectral width Δλ. Assuming that each wavelength has the same weight, the point spread function (*PSF*) of the optical system can be expressed by Equation (1), where $PSF_{m}(x,y,\lambda )$ is the *PSF* for the monochromatic light case, and it can be expressed by Equation (2), where B is the distance between the centers of the two sub-pupils, $J_{1}(\cdot )$ is the first order Bessel function, p is piston error, D is the diameter of the sub-pupil, and f is the focal length of the imaging lens.

(1) $$PSF_{b}(x,y,\lambda )=2\int_{\lambda_{0}-\frac{\Delta \lambda }{2}}^{\lambda_{0}+\frac{\Delta \lambda }{2}}PSF_{m}(x,y,\lambda )d\lambda$$

(2) $$PSF_{m}(x,y,\lambda )=2\frac{\frac{D^{2}}{2}J_{1}^{2}(\pi D\sqrt{{\left(\frac{x}{\lambda f}\right)}^{2}+{\left(\frac{y}{\lambda f}\right)}^{2}})}{\sqrt{{\left(\frac{x}{\lambda f}\right)}^{2}+{\left(\frac{y}{\lambda f}\right)}^{2}}}\times [1+cos\frac{2\pi }{\lambda }(p-\frac{B}{f}x)]$$

The integral of Equation (1) can be approximated as a differential summation by equating the range of the integral into n intervals, and it can be expressed by Equation (3).

(3) $$PSF_{b}(x,y,\lambda )=\sum_{i=1}^{n}\left[\frac{PSF_{m}(x,y,\lambda_{i})\Delta \lambda }{n}\right]=\frac{\Delta \lambda }{n}\sum_{i=1}^{n}\left\{\begin{array}{l} 2\frac{\left(\frac{D^{2}}{2})f^{2}\lambda_{i}^{2}J_{1}^{2}(\frac{\pi D}{\lambda_{i}f}\sqrt{x^{2}+y^{2}}\right)}{x^{2}+y^{2}}\times \\ \left[1+cos\frac{2\pi }{\lambda_{i}}(p-\frac{B}{f}x)\right] \end{array}\right\}$$

The *MTF* can be expressed by Equation (4), where $MTF_{sub}$ is the *MTF* of a single-aperture diffraction-limited imaging system with aperture D, $f_{x}=x_{0}/\lambda f$, $f_{y}=y_{0}/\lambda f$. $\rho =\sqrt{f_{x}^{2}+f_{y}^{2}}$. If $\rho \geq \frac{D}{\lambda f}$, $MTF_{sub}=0$, else the $MTF_{sub}$ is shown in Equation (5).

As shown in Equation (4), the *MTF* consists of a center peak and two side lobes. The center normalized amplitude of the *MTF* side lobe ($MTF_{nph}$) decreases with the increase in piston error, and the amplitude will be zero when the value of piston error reaches the coherence length. The coherence length L can be expressed by Equation (6), so the method can break the ambiguity of 2π to extend the capture range to the coherence length. The relationship between the $MTF_{nph}$ and the piston error is expressed by Equation (7).

(4) $$\begin{array}{l} MTF(f_{x},f_{y})=\left|FT\right.\left.\left[PSF_{b}(x,y,\lambda )\right]\right|=2MTF_{sub}(f_{x}+f_{y})+\\ \left[MTF_{sub}(f_{x}-B/\lambda f,f_{y})+MTF_{sub}(f_{x}-B/\lambda f,f_{y})\right] \end{array}$$

(5) $$MTF_{sub}=\frac{2}{\pi }\left(\arccos(\frac{\lambda f}{D}\rho )-(\frac{\lambda f}{D}\rho )\sqrt{1-{\left(\frac{\lambda f}{D}\rho \right)}^{2}}\right)$$

(6) $$L=\lambda_{0}^{2}/\Delta \lambda$$

(7) $$MTF_{nph}=\frac{1}{n}\sqrt{n+2[\sum_{j=1}^{n-1}\sum_{i=j}^{n-1}cos(\frac{2\pi }{\lambda_{j}}p-\frac{2\pi }{\lambda_{i+1}}p)]}$$

If a multi-aperture mask is placed in a multi-sub-mirror segmented optical system and the position of the sub-mirror No. 1 is used as a reference, the above size mapping relationship in Equation (7) remains correct as long as the *MTF* of the optical system is a non-redundant *MTF* [19,20,21]. Therefore, the magnitude of each $MTF_{nph}$ can effectively represent the piston error of each sub-mirror. 

**Figure 2:**
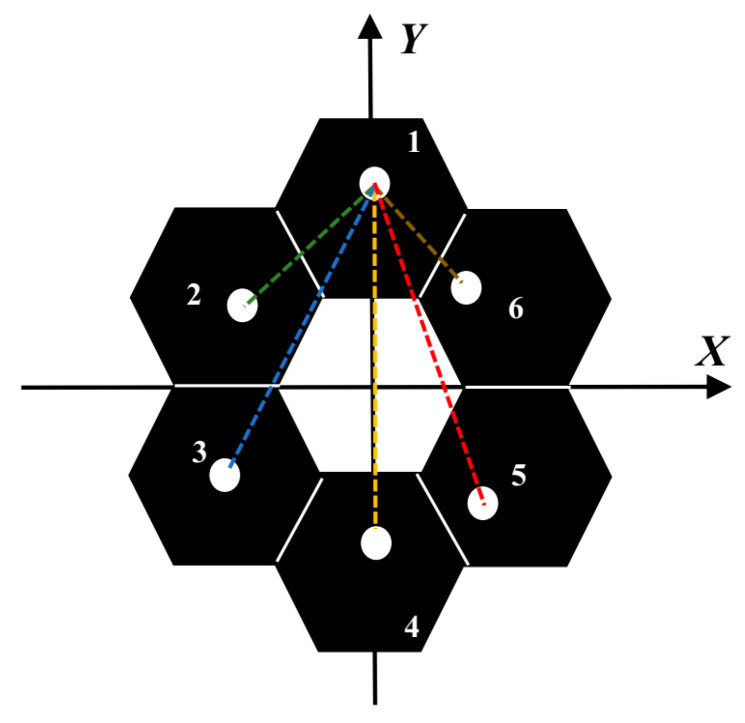
The schematic diagram of the segmented mirror structure and the configuration of the 6 sub-pupil masks.

We follow the method in the reference [15,16,17] to design the corresponding mask. The interference baseline between the measuring sub-pupil and the reference sub-pupil is called the main baseline, and the interference baseline between the measuring sub-pupils is called the sub-baseline. The schematic diagram of the mask is shown in Figure 2, where the position of sub-mirror No. 1 is taken as the reference, and the dashed line of each color is the interference main baseline of each sub-mirror and sub-mirror No. 1, respectively. The design principles of the mask are as follows: the number of sub-pupils is the same as the number of sub-mirrors, and each sub-pupil captures the reflected light of the corresponding sub-mirror and has the same size. The length difference between each two main baselines should be greater than the diameter of the sub-pupil, or the angle between them should be greater than θ, and the sub-baseline should also conform to similar conditions [17], where θ is the angle between the two main baselines when the center distance between two measuring sub-pupils is equal to the diameter of the sub-pupil.

**Figure 3:**
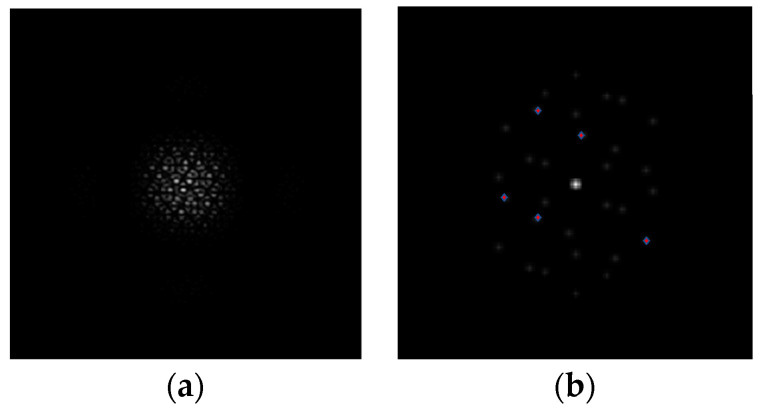
The *PSF* (**a**) and the *MTF* (**b**) of the segmented mirror with the mask.

The *PSF* and *MTF* of the corresponding optical system are shown in Figure 3. The red star points in Figure 3b are the $MTF_{nph}$ collected, which is used to reflect the piston errors of each sub-mirror. 

**Figure 4:**
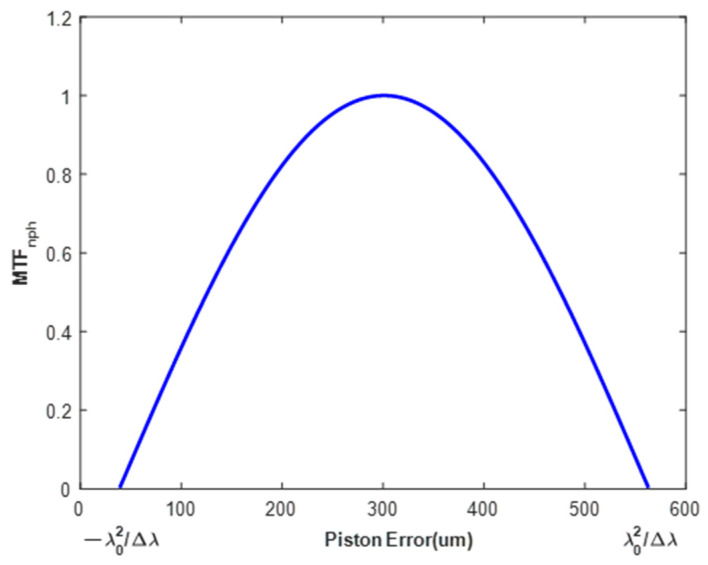
The diagram of the relationship between the piston error of a sub-mirror and the corresponding normalized $MTF_{nph}$ value.

The relationship between normalized $MTF_{nph}$ of a sub-mirror and the piston error of it can be calculated according to Equation (6), as shown in Figure 4. 

It can be seen from Figure 4 that $MTF_{nph}$ monotonically decreases as the piston error increases. When there are piston errors, tip errors, and tilt errors in the sub-mirrors, the monotonicity of this relationship no longer exists, and the relationship will become very complicated. $MTF_{nph}$ But the relationship of only when all the co-phase errors are zero that the $MTF_{nph}$ takes a maximum value still exists [19]. This specific relation is not required as the basis of the algorithm in this paper, so this relation is not explained in detail. The optimization algorithm based on population has been widely used in solving this kind of problem because it does not require continuity, convexity, conductibility, and connectivity of feasible domain. Therefore, we can construct the evaluation function based on this relation and realize the large range and high precision detection of piston error, tip error, and tilt error of each sub-mirror based on the population optimization algorithm. 

The incident light is reflected through the primary mirror into the optical system, and the optical path difference is twice the actual longitudinal displacement of the two sub-mirrors along the optical axis. So, the co-phase error correction range of the sub-mirror can be set as $\left[-\lambda_{0}^{2}/(2*\Delta \lambda ),\lambda_{0}^{2}/(2*\Delta \lambda )\right]$ and the 1/2 factor is introduced. At the same time, due to the special design of the mask, the interference baselines of each sub-mirror and the reference mirror do not coincide, so this method will not increase the difficulty of solving due to the increase in the number of sub-mirrors.

Below, we will explain the method and algorithm flow of constructing the evaluation function based on $MTF_{nph}$.

## 3. Phasing Three Dimensional Co-Phase Error in Segmented Telescopes by Mask

### 3.1. The Cuckoo Search Algorithm

The Cuckoo Search algorithm (CS) is a swarm intelligence algorithm [22,23,24], which was proposed by Suash Deb and Xin-She Yang in 2009. In recent years, due to its advantages, such as easy implementation, few parameters, and simple model, it has been widely used in various numerical optimization problems.

The CS algorithm simulates the principle of parasitic propagation of a Cuckoo’s nest and utilizes the Levy Flight mechanism to solve optimization problem effectively. 

The position update formula of the CS algorithm based on the Levy flight mechanism can be expressed by the following Equation (8), where the Levy flight can be simulated using the Mantegna method [25], which can be expressed by the following Equation (9).

In Equation (8), $x_{i}$ and $x_{i+1}$ are the host nest locations of the i and i+1 generations, $x_{best}$ is the current optimal solution, ⊕ is the point-to-point multiplication, Levy(*λ*) is the Levy flight formula, and a is the step control factor. In this paper, we use variable step size CS algorithm [17], the value of a can be calculated from Equation (10), where t is the current number of iterations, $T_{\max}$ is the maximum number of iterations.

(8) $$x_{i+1}=x_{i}+\alpha \left(x^{k}-x_{best}\right)⊕Levy(\lambda )$$

(9) $$Levy(\lambda )=\frac{\mu }{{\left|\nu \right|}^{1/\beta }}$$

(10) $$a=\exp(-30\times {\left(t/T_{\max}\right)}^{p}).$$

where $u∼N(0,\sigma_{u}{}^{2}),\nu ∼N(0,\sigma_{\nu }{}^{2}).$ The standard deviation $\sigma_{u}$ of the normal distribution is calculated according to Equation (11).

(11) $$\sigma_{u}={\left\{\frac{\Gamma (1+\beta )\sin(\pi \beta /2)}{\Gamma [(1+\beta )/2]\beta 2^{(\beta -1)/2}}\right\}}^{1/\beta },\sigma_{\nu }=1.$$

The probability that a host bird finds a parasitic egg is called the discovery probability $p_{a}$, which is usually equal to 0.25. CS algorithm generates a random number $r\in \left[0,1\right]$ after updating the position by Levy flight. When $r>p_{a}$, it randomly changes $x_{i}$ and generates an equal amount of new solutions to replace the old ones by means of preference walking, symbolizing the behavior of the host to find and dispose of the parasitic eggs, so the cuckoo randomly selects a new nest for parasitic reproduction. The preference wandering formula is shown in Equation (12), where the compression factor η∼U(0,1), and $x_{i}^{a},x_{i}^{b}$ represents two random solutions of the i generation.

(12) $$x_{i+1}=x_{i}+h\left(x_{i}^{a}-x_{i}^{b}\right)$$

**Figure 5:**
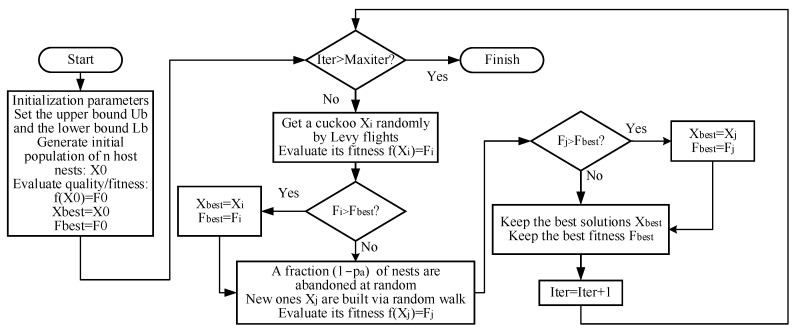
The flow chart of the CS algorithm.

When dealing with the actual optimization problem, the location of the nest represents the solution value of the variable to be identified, and the fitness of the nest represents the corresponding objective function value where the variable to be identified takes different solutions. The flow chart of the CS algorithm is shown in Figure 5 below:

### 3.2. The Algorithm Flow of Phasing Three Dimensional Co-Phase Error 

In this article, we consider two scenarios:

1. The optical system model has been accurately modeled;
2. The optical system is not accurately modeled.

Both cases are explained in detail below.

#### 3.2.1. The Algorithm Flow with Accurate Optical System Model

In the case that the optical system model has been accurately modeled, we can bring the solution generated by the CS algorithm into the optical system model so as to calculate its corresponding $MTF_{nph}$ value. This value will be marked as $MTF_{nph}{}_{-i}$. At this time, the $MTF_{nph}$ value corresponding to the real co-phase error of a sub-mirror is marked as $MTF_{nph}{}_{-T}$. According to the maximum likelihood theory [26], the corresponding evaluation function expression is constructed as $f(i)={\left|MTF_{nph}{}_{-i}-MTF_{nph}{}_{-T}\right|}^{2}$, which is similar to the phase diversity algorithm [17]. The rest of the algorithm flow is the same as in Figure 5.

But in the simulation experiment, we find that the influence of the three co-phase errors of piston, tip, and tilt on $MTF_{nph}$ can offset each other to a certain extent, and there are many local minima similar to the global minima in the optimization space of $f(i)={\left|MTF_{nph}{}_{-i}-MTF_{nph}{}_{-T}\right|}^{2}$. Therefore, although the dimension of the optimization space is only 3, the above algorithms are basically not convergent, and eventually, they will fall into the local extreme value and fail to find the global optimal value.

#### 3.2.2. The Algorithm Flow without Accurate Optical System Model

In order to solve the above problem, we use the thought of model-free online correction to improve the evaluation function in the above method.

The method of the model-free online correction does not rely on the environment model, directly obtains empirical data via the interaction with the environment, and learns and optimizes according to these data, which is a widely used concept in reinforcement learning algorithms [27,28]. 

Here, we no longer use $MTF_{nph}$ to calculate the co-phase error of the sub-mirror in reverse, but by correcting the co-phase error of the sub-mirror to be identified and then calculating the $MTF_{nph}$ value corresponding to the residual co-phase error to evaluate the quality of the correction amount taken. When the adopted sub-mirror correction amount is exactly the same as the actual sub-mirror co-phase error value, that is, the residual co-phase error of the sub-mirror is zero, the $MTF_{nph}$ corresponding to the residual co-phase error will take the maximum value. The sub-mirror correction amount will be generated by the CS algorithm. As described above, the input of the evaluation function will become $\left(c_{1}-c_{1i},c_{2}-c_{2i},c_{3}-c_{3i}\right)$, where $\left(c_{1i},c_{2i},c_{3i}\right)$ is a solution of the CS algorithm. Since the real co-phase error $c_{1},c_{2},c_{3}$ of the sub-mirror is unknown, this method requires real-time correction of the position of the existing sub-mirror according to the correction amount $\left(c_{1i},c_{2i},c_{3i}\right)$ to simulate the input amount $\left(c_{1}-c_{1i},c_{2}-c_{2i},c_{3}-c_{3i}\right)$ and then calculate the $MTF_{nph}$ as the evaluation function value.

In this way, the co-phase error value is no longer calculated in reverse depending on the $MTF_{nph}$ value. Therefore, the influence of local extreme values on the optimization algorithm will be reduced to some extent. In addition, this method does not rely on the exact relationship between $MTF_{nph}$ and co-phase error, so the algorithm failure caused by the difference between the simulated calculated value and the actual value can be effectively avoided and does not even need to model the optical system.

**Figure 6:**
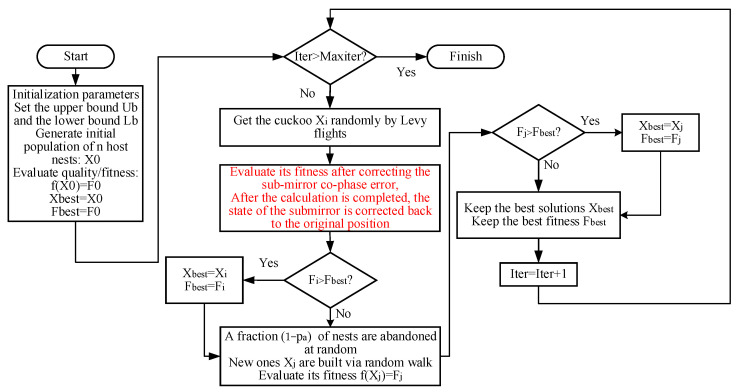
The algorithm flow of phasing three dimensional co-phase error in segmented telescopes by mask without accurate optical system model.

However, we do not know the actual co-phase error of the sub-mirror in the actual situation, and each solution in the population optimization algorithm needs to calculate its own evaluation function value. Therefore, the sub-mirror needs to be corrected to the original state after correcting according to each solution, so that the next solution in the population algorithm can be used to correct the position of the sub-mirror and obtain the relevant evaluation function value. Therefore, the number of individuals in the population of this method is not too large so as not to increase too many meaningless correction times. The algorithm flow is shown in Figure 6 below:

## 4. Simulation Experiment

We set up this optical system in MATLAB; the relevant parameters of the segmented optical mirror simulation system are as follows: the primary mirror consists of 6 hexagon sub-mirrors, the F# of the optical system is 10, the CCD pixel size is 2.5 µm, the exit pupil plane size to 256 × 256 pixels, the diameter of the circle on the mask is 8 pixels, the circumscribed circle diameter of the single hexagonal sub-mirror is 40 pixels, the mask is set according to the principle of reference [14]. 

The central wavelength of the spectrum in this paper is 632.8 nm, Δλ=6.328 nm and L=63,280 nm can be obtained according to Equation (5). Therefore, the correction range of each sub-mirror piston error is set to [−50λ,50λ] in this paper.

We randomly generate 100 groups of sub-mirror co-phase errors within [−50λ,50λ], and use the above method to correct each group of co-phase errors. The sub-mirror No. 1 is the standard, and sub-mirror No. 6 is the corrected sub-mirror. The population in the CS algorithm is 20, the maximum number of iterations in each optimization process is 500, and the parameters in the step size control factor p=30. The final results of each group of experiments are recorded, and the RMSE value is calculated by Equation (13), where $c_{i}^{t}$ are the *i*-th true aberration coefficients, and $c_{i}^{r}$ is the corresponding final correction aberration coefficients after 500 iterations. 

Since the $MTF_{nph}$ reflecting the co-phase errors of each sub-mirror are independent of each other, only the experimental results of sub-mirror No. 6 are given in this paper; the simulation results of other sub-mirrors are similar. However, in order to represent the effectiveness of the method, the three-dimensional co-phase errors of all sub-mirrors except for sub-mirror No. 1 are generated randomly within [−50λ,50λ].

(13) $$\mathrm{RMSE}={\left\{\frac{\sum_{i=1}^{3}{\left(c_{i}^{t}-c_{i}^{r}\right)}^{2}}{3}\right\}}^{1/2}.$$

**Figure 7:**
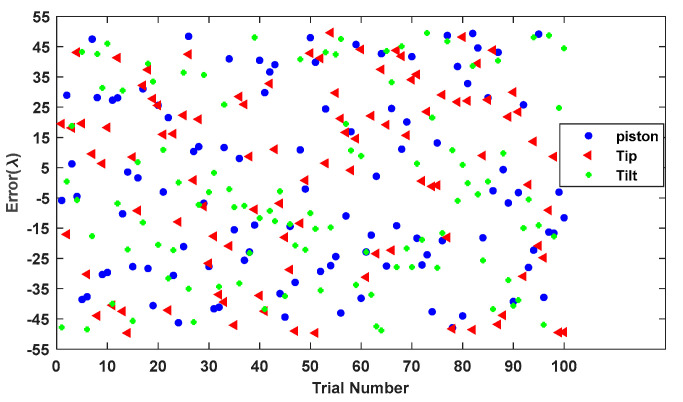
The piston error, tip error, and tilt error of 100 randomly generated sub-mirror No. 6 co-phase errors.

**Figure 8:**
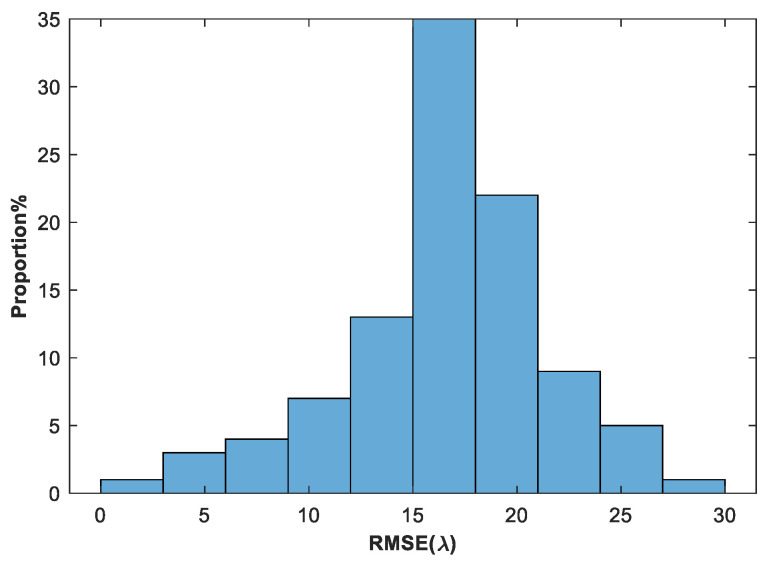
The statistical histogram of RMSE values of 100 randomly generated sub-mirror No. 6 co-phase errors.

Figure 7 shows the values of piston error, tip error, and tilt error among 100 groups of randomly generated sub-mirror No. 6 co-phase errors. Figure 8 shows the statistical histogram of co-phasing error RMSE values for the 100 groups.

**Figure 9:**
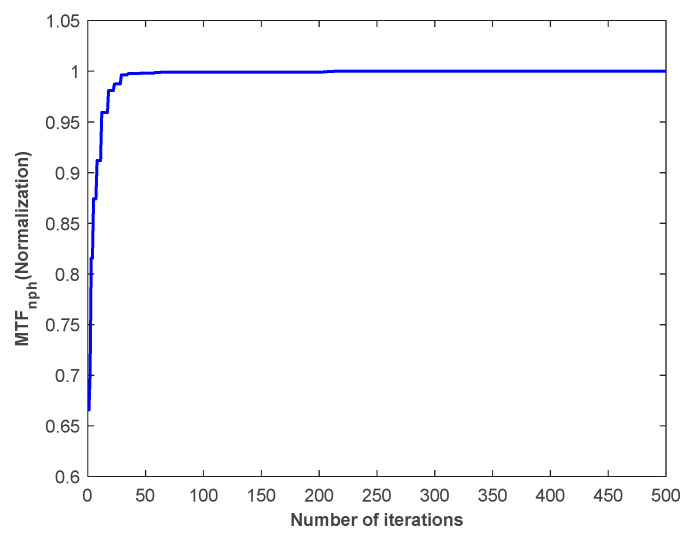
The change curve of normalized $MTF_{nph}$ value with the number of algorithm iterations in a typical experiment of sub-mirror No. 6.

**Figure 10:**
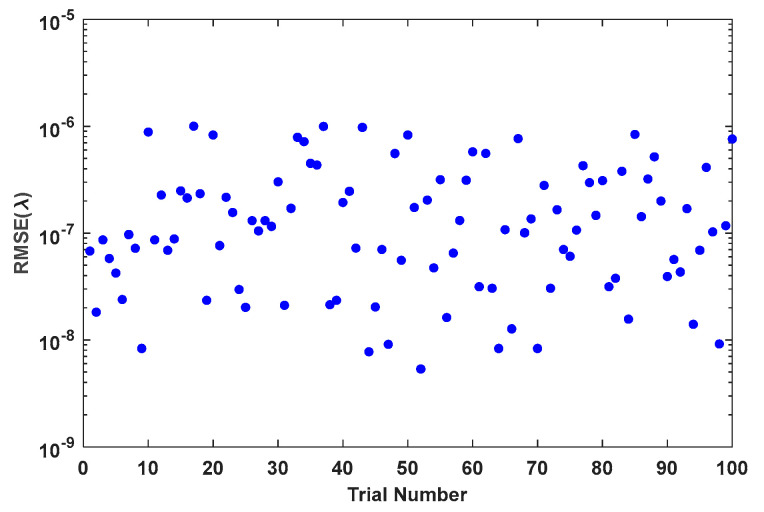
The RMSE of the true coefficients and the corresponding final correction aberration coefficients after 500 iterations of the 100 experiments.

**Figure 11:**
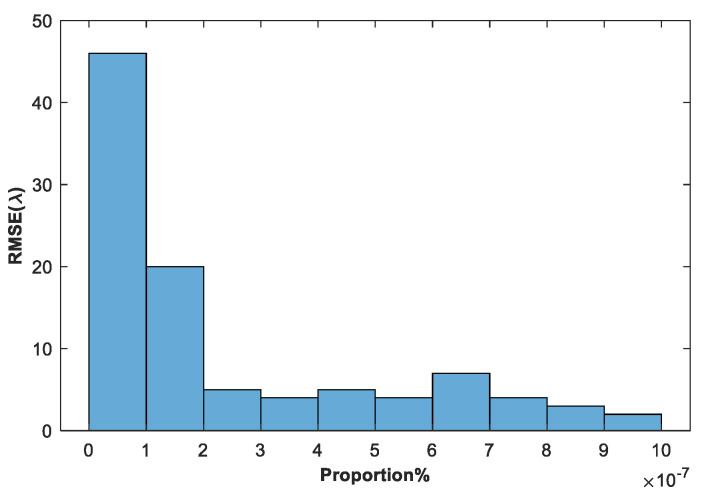
The statistical histogram of RMSE of the true coefficients and the corresponding final correction aberration coefficients after 500 iterations of the 100 experiments.

Figure 9 shows the change curve of normalized $MTF_{nph}$ value with the number of algorithm iterations in a typical experiment of sub-mirror No. 6. Figure 10 shows the RMSE of the true coefficients and the corresponding final correction aberration coefficients after 500 iterations of the 100 experiments. Figure 11 shows the statistical histogram of RMSE of the true coefficients and the corresponding final correction aberration coefficients after 500 iterations of the 100 experiments.

Experimental results show that the proposed method can effectively sensing and correct the co-phase errors of the sub-mirrors in the range of [−50λ,50λ] with high precision, and the average RMSE value of 100 experiments is 2.358 × 10^−7^*λ*.

Compared to other iteration-based methods, this method takes less time because the optimization dimension of this method is only 3. In addition, because the identification and correction of the co-phase errors of each sub-mirror are independent of each other, they can be carried out simultaneously, avoiding the problem that the difficulty of calculation and the solving time increase exponentially with the increase in the number of sub-mirrors. While this approach takes longer than non-iterative deep learning-based approaches, it does not require accurate modeling of the optical system. In the actual optical system, the accuracy mainly depends on the displacement resolution of the high-precision displacement adjusting mechanism used to adjust the position of the sub-mirrors. Therefore, although other methods may have higher recognition accuracy, the final correction accuracy also depends on the high precision displacement adjusting mechanism, so the final correction accuracy will be comparable to the method in this manuscript.

When the number of sub-mirrors is large, by grouping the segment, the method can still be effective, no matter the aperture and geometry of the primary mirrors [17].

## 5. Conclusions

In this paper, an independent co-phase error sensing and correction method for each sub-mirror of the segmented mirror is proposed based on mask and wide-spectrum *MTF*. The sub-peak value of wide-spectrum *MTF* can reflect the magnitude of the co-phase error of the sub-mirror to a certain extent. This method is based on the sub-peak value of wide-spectrum *MTF* to build a corresponding evaluation function. And after correcting the co-phase error of the sub-mirror by real-time correction method, the reward function is calculated to judge the quality of each solution in the cuckoo search optimization algorithm. Therefore, it does not depend on the specific relationship between the *MTF* secondary peak value of the wide spectrum and the sub-mirror co-phase error but only needs to maintain the size mapping relationship between the two. The method even does not need to model the optical system. This feature increases the robustness of the algorithm and avoids the risk of algorithm failure caused by the difference between the simulation model and the actual model. Meanwhile, this method can correct the three co-phase errors of the piston, tip, and tilt of the sub-mirror at the same time. Since the co-phase error correction of each sub-mirror is independent of each other, this method is not limited by the number of sub-mirrors. Increasing the number of sub-mirrors does not make the algorithm more difficult, and we only need to design the corresponding mask according to the rules. And the efficiency is high because the co-phase error correction process of each sub-mirror can be corrected simultaneously. Simulation results show that the proposed method can effectively sensing and correct the co-phase errors of the sub-mirror s in the range [−50λ,50λ] of three dimensions with high precision. The average RMSE value of one of the six sub-mirrors in 100 experiments is 2.358 × 10^−7^λ. In the actual optical system, the accuracy mainly depends on the displacement resolution of the high-precision displacement adjusting mechanism used to adjust the position of the sub-mirror. In the future, we will conduct further experiments to continue the research of the method in this paper. 

## Data Availability

The data are not publicly available due to privacy.
